# Efficient Generation of Microdroplets Using Tail Breakup Induced with Multi-Branch Channels

**DOI:** 10.3390/molecules26123707

**Published:** 2021-06-17

**Authors:** Daiki Tanaka, Satsuki Kajiya, Seito Shijo, Dong Hyun Yoon, Masahiro Furuya, Yoshito Nozaki, Hiroyuki Fujita, Tetsushi Sekiguchi, Shuichi Shoji

**Affiliations:** 1 Research Organization for Nano & Life Innovation, Waseda University, 513 Wasedatsurumakicho, Shinjuku-ku, Tokyo 162-0041, Japan; yoon@shoji.comm.waseda.ac.jp (D.H.Y.); y.nozaki@aoni.waseda.jp (Y.N.); t-sekiguchi@waseda.jp (T.S.); 2 Department of Electronic and Physical Systems, School of Fundamental Science and Engineering, Waseda University, 3-4-1 Okubo, Shin-juku-ku, Tokyo 145-0065, Japan; kajiya@shoji.comm.waseda.ac.jp (S.K.); shijo@shoji.comm.waseda.ac.jp (S.S.); furuya@aoni.waseda.jp (M.F.); shojis@waseda.jp (S.S.); 3 Canon Medical Systems Corporation, 1385 Shimoishigami, Otawara-shi, Tochigi 324-8550, Japan; hiroyuki12.fujita@medical.canon

**Keywords:** microfluidics, single-micrometer scale, droplet generation, encapsulation

## Abstract

In recent years, research on the application of microdroplets in the fields of biotechnology and chemistry has made remarkable progress, but the technology for the stable generation of single-micrometer-scale microdroplets has not yet been established. In this paper, we developed an efficient and stable single-micrometer-scale droplet generation device based on the fragmentation of droplet tails, called “tail thread mode”, that appears under moderate flow conditions. This method can efficiently encapsulate microbeads that mimic cells and chemical products in passively generated single-micrometer-scale microdroplets. The device has a simple 2D structure; a T-junction is used for droplet generation; and in the downstream, multi-branch channels are designed for droplet deformation into the tail. Several 1–2 µm droplets were successfully produced by the tail’s fragmentation; this continuous splitting was induced by the branch channels. We examined a wide range of experimental conditions and found the optimal flow rate condition can be reduced to one-tenth compared to the conventional tip-streaming method. A mold was fabricated by simple soft lithography, and a polydimethylsiloxane (PDMS) device was fabricated using the mold. Based on the 15 patterns of experimental conditions and the results, the key factors for the generation of microdroplets in this device were examined. In the most efficient condition, 61.1% of the total droplets generated were smaller than 2 μm.

## 1. Introduction

Droplet downsizing is attracting attention as an important technology for biomedical and chemistry applications. In biology, encapsulating a target cell with a droplet enables gene analysis using polymerase chain reaction [1,2] and observing the behavior of the individual cell dynamically, such as specific biochemical reactions [3,4]. Usually, droplets with a diameter of 10 µm or more are used, these being larger than the cells. However, smaller capsules have been required in recent years as the observed targets changed from cells to organelles. Droplet size reduction is also important in chemistry. Banerjee et al. [5] reported that the rates of chemical reactions in microdroplets were improved over those in beakers. A single-molecular enzymatic assay was achieved by containing a single molecule in a fL-chamber [6,7]. Our research group has also used microdroplets for microdropletization and organic chemical synthesis of various organic solvents [8,9]. Downsizing microdroplets is expected to improve reaction efficiency and product concentration in chemical synthesis.

Many researchers have studied droplet formation [10,11,12,13,14], and the well-known microfluidic device geometries are the T-junction and flow focusing, with droplets formed by flow focusing being smaller than those formed by a T-junction. In flow focusing, an immiscible continuous fluid flows coaxially around the dispersed fluid and squeezes the latter to generate droplets. There are five droplet formation modes [15,16,17], namely, squeezing, dripping, jetting, tip multi-breaking, and tip streaming. It has been reported that tip streaming generates the smallest droplets, so tip streaming is commonly used for producing microdroplets. Since Taylor first proposed the phenomenon [18], several studies have succeeded in generating submicron droplets by tip streaming. Martz et al. [19] successfully produced droplets smaller than 1 µm using a two-dimensional (2D) flow focusing device with tip streaming, but the latter was difficult to maintain, and stable generation of nanodroplets was not realized. Jeong et al. [20] and Xu et al. [21] fabricated a three-dimensional (3D) flow focusing device with improved stable tip streaming, but 3D device fabrication requires both manual dexterity and alignment under a microscope. Inducing tip streaming generally requires a high ratio of the flow rates of the continuous and dispersed fluids, and limited flow rate conditions make droplet applications difficult [22]. Some researchers have reported generating submicron droplets without using tip streaming. Malloggi et al. [23] succeeded in generating 900-nm droplets using step emulsification with a nanochannel, but nanoscale devices are difficult to fabricate and have high internal resistance. Márin et al. [24] and Wu et al. [25] generated submicron droplets without using microfluidic devices. However, microfluidic devices facilitate a series of experiments, from separation to observation, and are therefore preferred for bio-applications. Therefore, it is necessary to develop a device that realizes the stable and highly efficient generation of single-micrometer droplets (S-MD: femtoliter) with a simple design and passive microfluidics. The generation of S-MDs through the application of chip streaming causes damage to internal biological samples and chemical compounds.

This paper is a brief report on a new method to efficiently generate S-MDs using tailing in a simple device, and on the detailed experimental conditions. In addition, we demonstrated a technique to efficiently encapsulate microbeads that mimic cells and compounds into S-MDs.

## 2. Results and Discussion

### Device Design and Fabrication

**Figure 1:**
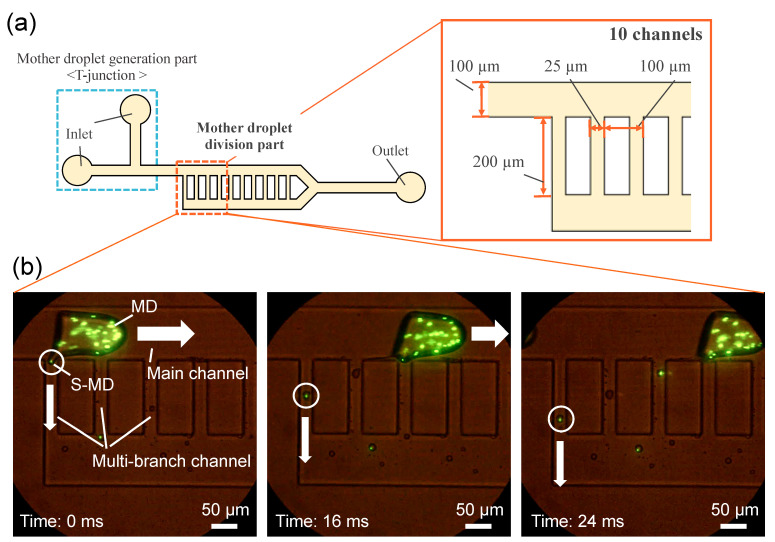
(**a**): Dimensions of device for generating microdroplets using tail breakup. (**b**): Inclusion of fluorescent microbeads. Microbeads in MoDs are successively encapsulated in S-MDs.

The dimensions of the device are shown in Figure 1a. A T-junction is used to generate the mother droplets (MoDs), and multi-branch channels are designed downstream so that the flow rates at the inlet and outlet of the division section are the same. The height of all the channels is 100 µm, and the channel width, except for the branch channels, is 100 µm. The branch channels are designed at 75 µm intervals, being 25 µm wide and 200 µm long. The mold for the device was fabricated by soft lithography, and polydimethylsiloxane (PDMS) was poured into the mold to be thermally cured before the PDMS device was used. Before the experiments, the PDMS device was baked at 80 °C for at least a week and then degassed for over an hour.

The S-MD is generated by a method of stretching and separating from an MoD. Specifically, the S-MD stretched from the MoD flowing in the main channel is split and separated in the multi-branch channel. The generated S-MD is then carried downward through the multi-branch channel. Figure 1b shows the results of an experiment in which fluorescent microbeads (particle size: 3 μm) were encapsulated to mimic cells and chemical compounds. The microbeads were encapsulated in the tailing portion that extended from the MoD. The microbeads were encapsulated efficiently in S-MDs, and the passive generation of the latter would enable efficient extraction of cells and chemical products in the MoD. The flow conditions for the fluid experiment were Tween 20 0.5 w% (dispersed flow) and Span 80 0.5 w% (continuous flow) + 10 μL/min.

The flow rate and interfacial tension (surfactant concentration) are key parameters for droplet generation; therefore, S-MD formation was observed while changing these two parameters. In the first experiment, only the continuous phase contained surfactant. Under this condition, no tailing was observed and the MoD did not split. Studies have been reported in which tip streaming was used to produce tiny droplets, and this requires surfactant in bulk [26,27]. Therefore, in the second experiment, both the continuous and dispersed phases contained surfactants. Table 1 shows the surfactant concentration and flow rate in the fluid experiments.

**Figure 2:**
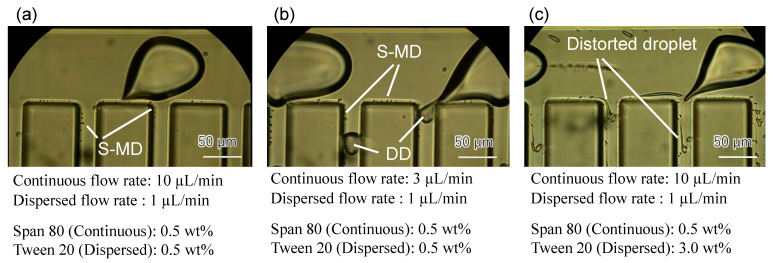
Generation of microdroplets under various conditions. (**a**): S-MDs are efficiently generated. (**b**): S-MDs and DDs are generated simultaneously. (**c**): The size and shape of S-MDs are not stable.

Figure 2 shows a typical result of the fluid experiment. In the condition of Figure 2a, only S-MDs are efficiently generated. A thin and narrow tail is extracted from an MoD and broke into S-MDs. In Figure 2b with the smaller flow rate of the continuous phase, not only S-MDs, but also daughter droplets (DD: direct partitioning of mother liquor) are generated. In Figure 2c with the increased amount of surfactant in the dispersed phase, the tail did not fully break into droplets and the droplet sizes are not uniform. The generation of S-MDs by the tailing of MoDs could not be achieved unless the surfactant was placed in both the continuous and dispersed flows. The generation of S-MDs is considered to require precise adjustment of the interfacial tension. Span 80 is insoluble in water and easily soluble in oil. On the other hand, Tween 20 has the opposite property. Therefore, different surfactants should be used.

The experimental results showed that DD generation decreased with increasing carrier flow rate; the most efficient conditions for S-MD generation were 0.5 wt% concentrations of Tween 20 and Span 80 and flow rates of 10 μL/min for continuous flow and 1 μL/min for dispersed flow.

**Figure 3:**
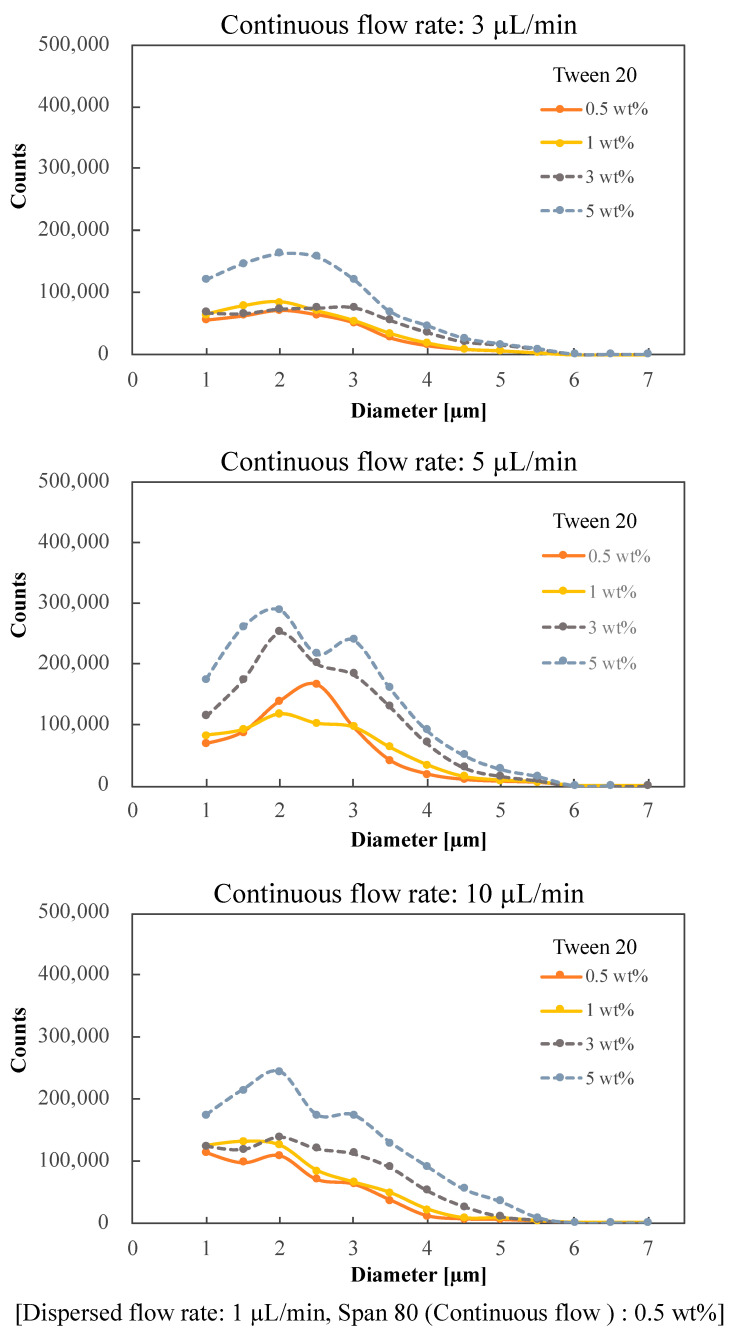
Number distribution of S-MDs with different surfactant concentrations and continuous flow rates in the dispersion flow.

Figure 3 shows the graphs for different concentrations of surfactant (Tween 20) added to the dispersion flow. The size of the S-MDs was calculated from the pixel display of the high-speed camera. The number was quantified using image recognition machine learning. At a continuous phase concentration of 0.5 wt%, the distribution of microdroplet diameters was concentrated between 1.0 and 3.0 μm, and the peak tended to shift toward 1 μm as the continuous phase flow rate increased. The number of S-MDs increased with the increase in continuous flow. When the flow rate of the continuous flow was 3 μL/min, the number of S-MDs was small and the variation was large.

When the concentration of Span 80 was set to 3 wt%, the size of the microdroplets varied due to the formation of a large number of satellite droplets (Supplementary Materials, Figure S1).

On the other hand, when the dispersed phase concentration was more than 3 wt%, the number of droplets generated was the largest at a continuous phase flow rate of 5 μL/min. This is probably due to the small number of satellite droplets (polydisperse droplets generated on the channel wall) generated when the flow velocity of the continuous phase is high. When the dispersed phase concentrations are higher than 3 wt%, satellite droplets are generated and the size variation of the microdroplets is large, which is not suitable for the generation of S-MDs (dashed line).

The optimum conditions for the generation of small droplets were found to be a surfactant concentration of 0.5 wt% in both phases and a flow rate of 10 μL/min in the continuous phase, which resulted in a 61.1% generation rate of small droplets less than 2 μm in total. The microdroplet generation rate of less than 2 μm was 61.0% when the concentration of Tween 20 was 1 wt% and the continuous flow rate was 10 μL/min. However, 0.5 wt% was set as the optimum condition because the concentration of surfactant should be as low as possible considering the use of S-MDs for cell culture and chemical reaction.

Table 2 shows the viscosity of the solution for each surfactant concentration.

**Figure 4:**
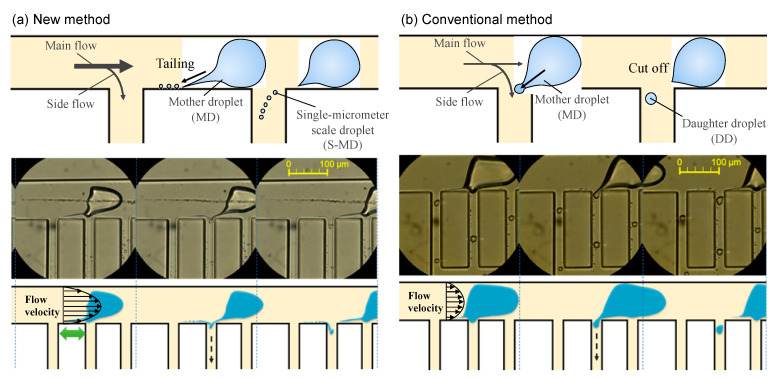
New method for generating single-micrometer droplets (S-MDs). (**a**) New method: MoD tailing occurs at the channel wall, and S-MDs are generated one after another; (**b**) Conventional method: Since the tail of MoD is cut off at the corner of the side channel, a DD is generated, and an S-MD is not generated.

Figure 4 compares our new method of generating S-MDs with the conventional method. In the new method (Figure 4a), the S-MD is generated by splitting a thin line from the MoD. In contrast, conventional droplet division caused by shear forces generates a DD, larger than the S-MDs, from the MoD (Figure 4b) [28].

Figure 4a shows that tailing is often observed during droplet formation and along the microchannel wall, and S-MDs produced by the tail splitting are reported to be three orders of magnitude smaller than the main droplet [23,24,25]. Because tailing results from the stretching of the droplet surface, for the tail to occur, it is necessary to use liquids with low interfacial tension and ensure that forces act in opposing directions so that the tail is elongated. We propose a simple 2D structure to cause the tail by branching the flow of the main channel using multi-branch channels, aiming to produce a large number of S-MDs by tail break-off. As shown in Figure 4a, an MoD is partially pulled by the side flow upon reaching a branch channel, and two different directional flows stretch it. The droplet tail is then formed by the strong and continuous fluid pull and splits into S-MDs. Furthermore, S-MDs are generated continuously by the serial side flow, which allows high-throughput production. In addition, the flow rate in the main channel is important. Figure 4a shows when the flow velocity of the continuous phase is high, the tail is considered to be stretched because of the larger difference in velocity between near the wall and near the center of the flow channel.

The principle behind the formation of S-MDs is thought to be the influence of the Plateau–Rayleigh instability and the Kelvin–Helmholtz instability. The Plateau–Rayleigh instability is the property that when a liquid is injected in the form of a rod, it splits into droplets as it moves forward due to surface tension [29,30]. The Kelvin–Helmholtz instability, on the other hand, is the formation of a velocity shear layer in the boundary region between the two fluids when they are in contact with each other at a relative velocity. In this boundary region, it is known that the motion is unstable to disturbances [31]. In addition, various factors such as the surface condition of the device and the interfacial tension of the solution may contribute to the generation of S-MDs.

## 3. Materials and Methods

### Experimental Setup and Materials

All solutions were introduced from inlets with syringes (1750CX, Hamilton, NV, USA) and syringe pumps (KDS210, kdScientific, Holliston, MA, USA). The experimental results were obtained using an optical microscope (IX71, OLYMPUS, Tokyo, Japan) and a high-speed camera (FASTCAM Mini AX, Photron, Tokyo, Japan). Mineral oil (M8410, CAS 8042-47-5, Sigma-Aldrich, St. Louis, MI, USA) with Span 80 (Cat. No. 37408-32, CAS 1338-43-8, Kanto Chemical, Tokyo, Japan) was used for the continuous flow, and deionized water with Tween 20 (P1379, CAS 9005-64-5, Sigma-Aldrich) was used for the dispersed flow. Surfactants were dissolved in both solutions.

## 4. Conclusions

In this study, we provide a simple but efficient S-MD generation method. The generation rate of S-MDs smaller than 2 μm was 61.1% in total. Furthermore, we applied this technology to develop a microdevice that can be used for single cell encapsulation and chemical product concentration. We successfully produced S-MDs using tailing and demonstrated the availability of the new device without a high flow rate. For use in applications, the device must be improved and optimized with analyzed requirements in the next step. Summarizing the experimental results obtained from the combination of oil flow rates and surfactant concentrations, the following are key factors for generating 1-µm droplets using the velocity difference from the wall surface: (1) the flow rate in the main channel determines the flow velocity gradient that keeps the tip of the tail near the wall surface of the low-velocity region and keeps the base of the tail away from the wall surface and stretches it thinly in the high-velocity region; (2) the surfactants reduce the interfacial tension, thereby elongating the tail and keeping it thin and undivided. These results and analyses contribute significantly to the fabrication of devices for S-MD applications. Here, we proposed a method for generating S-MDs, known as the tail thread mode by tailing, and analyzed the factors of S-MD formation to explore the usefulness of the new method.

## Figures and Tables

**Table 1:** Surfactant concentration and flow rate.

Surfactant Concentration (wt%)						Flow Rate (µL/min)
Tween 20	0.5	1.0	3.0	5.0	0.5	Dispersed: 1
Span 80	0.5	0.5	0.5	0.5	3.0	Continuous: 3
Tween 20	0.5	1.0	3.0	5.0	0.5	Dispersed: 1
Span 80	0.5	0.5	0.5	0.5	3.0	Continuous: 5
Tween 20	0.5	1.0	3.0	5.0	0.5	Dispersed: 1
Span 80	0.5	0.5	0.5	0.5	3.0	Continuous: 10

**Table 2:** Viscosity of surfactant solutions for different concentrations.

	Surfactant Concentration (wt%)			
	0.5	1	3	5
Water + Tween 20 (mPa·s)	0.8788	0.8788	1.014	1.128
Oil + Span 80 (mPa·s)	24.51	24.69	25.31	26.31

## Supplementary Materials

The following are available online, Figure S1 shows the graphs for different concentrations of surfactant (Span 80) added to the continuous flow. When the continuous phase concentrations are higher than 3 wt%, satellite droplets are generated and the size variation of the microdroplets is large, which is not suitable for the generation of S-MD (Dashed line).
