# Supplementary material for: Efficient Generation of Microdroplets Using Tail Breakup Induced with Multi-Branch Channels
Source: Molecules. 2021 Jun 17;26(12):3707. doi: 10.3390/molecules26123707 (PMC8235478; doi:10.3390/molecules26123707)
Supplement: Supplementary file 1 [file molecules-26-03707-s001.zip › molecules-1221810-supplementary.pdf]

# Supplementary Materials

# Efficient Generation of Microdroplets Using Tail Breakup Induced with Multi-Branch Channels


**Daiki Tanaka [1,*], Satsuki Kajiya [2], Seito Shijo [2], Dong Hyun Yoon [1], Masahiro Furuya [2], Yoshito Nozaki [1], Hiroyuki Fujita [3], Tetsushi Sekiguchi [1] and Shuichi Shoji [2]**

[1] Research Organization for Nano & Life Innovation, Waseda University, 513 Wasedatsurumakicho, Shinjuku-ku, Tokyo 162-0041, Japan; yoon@shoji.comm.waseda.ac.jp (D.H.Y.); y.nozaki@aoni.waseda.jp (Y.N.); t-sekiguchi@waseda.jp (T.S.)

[2] Department of Electronic and Physical Systems, School of Fundamental Science and Engineering, Waseda University, 3-4-1 Okubo, Shin-juku-ku, Tokyo 145-0065, Japan; kajiya@shoji.comm.waseda.ac.jp (S.K.); shijo@shoji.comm.waseda.ac.jp (S.S.); furuya@aoni.waseda.jp (M.F.); shojis@waseda.jp (S.S.)

[3] Canon Medical Systems Corporation, 1385 Shimoishigami, Otawara-shi, Tochigi 324-8550, Japan; hiroyuki12.fujita@medical.canon

* Correspondence: d.tanaka@ruri.waseda.jp; Tel.: +81-3-3204-5765

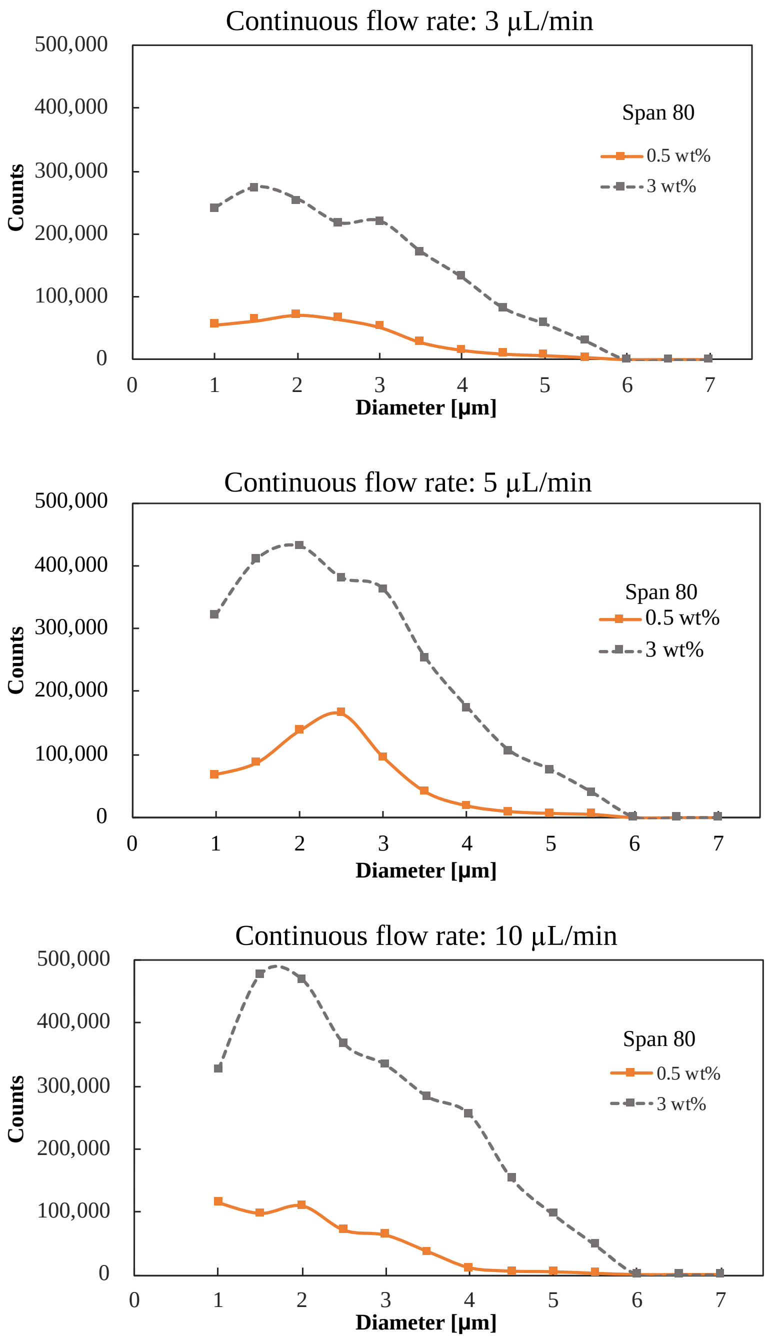


**Figure S1.** Varying the concentration of surfactant in the continuous flow.
